# Adjuvant skin-sparing electrochemotherapy in a breast cancer patient with a prosthetic implant: 5-year follow-up outcomes

**DOI:** 10.1093/jscr/rjac199

**Published:** 2022-05-18

**Authors:** Luca G Campana, Nicola Balestrieri, Nicola Menin

**Affiliations:** Department of Surgery, The Christie NHS Foundation Trust, Manchester, UK; Department of Surgery, Manchester Royal Infirmary, Manchester University NHS Foundation Trust, Manchester, UK; Breast Unit, ULSS 2 Marca Trevigiana, Vittorio Veneto, Italy; Breast Unit, ULSS 2 Marca Trevigiana, Vittorio Veneto, Italy

## Abstract

A 55-year-old woman with previous skin-sparing mastectomy and prosthetic reconstruction for multifocal ductal carcinoma developed homolateral axillary recurrence. Following nodal dissection, partial periprosthetic capsulectomy and the overlying breast skin excision, the pathology report revealed a positive cutaneous margin. Since further breast skin excision or radiotherapy would have compromised the prosthetic implant, and the patient was adamant about avoiding any endangering intervention, the multidisciplinary recommendation included skin-directed electrochemotherapy (ECT) in the frame of a multimodal treatment strategy. The procedure lasted 20 minutes under mild general sedation and included a bolus of intravenous bleomycin followed by local application of electric pulses using a needle electrode. The postprocedural course was uneventful, except for mild dermatologic toxicity. At 5 years, the patient is disease-free with the implant *in situ*. This report illustrates the proof-of-concept of adjuvant skin-sparing ECT to sterilize resection margins, preserve a breast implant and highlight procedural details to avert toxicity.

## INTRODUCTION

**Figure 1:**
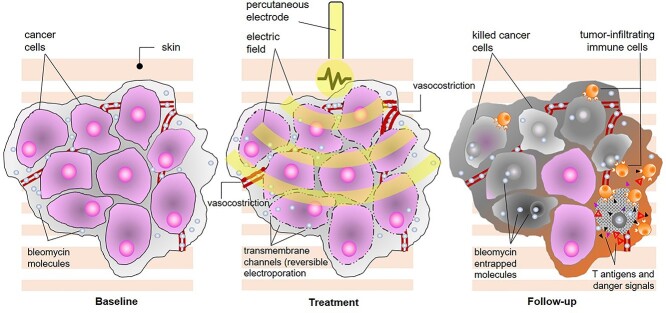
Overview of mechanism of action of electrochemotherapy. The procedure combines the injection or infusion of a cytotoxic drug (bleomycin or cisplatin) with the local application of short, high-voltage electric pulses, and the combined result is the enhancement of chemotherapy diffusion into the tumour thanks to the transient opening of aqueous pores on the cell membrane (reversible electroporation); since dividing cells are sensitive to chemotherapy, ECT provides the ability to selectively kill tumour cells (predominantly by drug-induced double-strand DNA breaks and apoptosis, although some necrosis also occurs) without harming normal surrounding tissue, and additional mechanisms of action include immediate local vasoconstriction (reduced drug washout), delayed endothelial disruption (leading to lack of oxygen and nutrients) and immune response to released antigens and danger-associated molecular patterns.

Breast cancer patients are exposed to skin tumour involvement in various forms along their journey, thus facing the peril of complex therapeutic decisions and debilitating interventions. Among the available skin-directed approaches, electrochemotherapy (ECT) combines a cytotoxic agent with short electric pulses. Based on temporary permeabilization (reversible electroporation) to chemotherapy, ECT produces a robust antitumour effect, whose underpinning mechanisms include direct cytotoxicity, a multifaceted vascular disrupting action and immune response [1–4] (Fig. 1). Cutaneous chest wall recurrences account for most of the current ECT indications in breast cancer. Summary data from initial experiences and, more recently, from publications between 2004 and 2019 indicate an overall and complete response rate of 74 and 46%, respectively [5, 6]. Although primarily employed in the palliative setting to control the growth of skin metastases following mastectomy, novel promising indications continue to emerge. In this regard, the adjuvant setting is uncharted territory. This report presents the first application of ECT in the adjuvant setting, following the marginal resection of a locoregional recurrence of breast cancer.

## CASE REPORT

A 55-year-old woman with a 6-year history of high-grade multifocal ductal carcinoma *in situ* treated with a skin-sparing mastectomy, immediate prosthetic reconstruction and sentinel lymph node biopsy came to our attention for homolateral axillary adenopathy. ECOG performance status was zero, with no significant comorbidities. She underwent axillary dissection and, given the advanced tumour infiltration, partial periprosthetic capsulectomy and excision of the overlying skin close to the tumour. The pathology report revealed a poorly differentiated ductal invasive carcinoma (hormone receptors positive, human epidermal growth factor receptor 2 [HER2]-negative, ki67 40%) in 2 out of 18 lymph nodes and microscopic tumour deposits on the periprosthetic capsule and the breast skin, the latter with a positive resection margin. A wider skin excision or radiotherapy would have likely compromised the prosthetic implant, and the patient was adamant about avoiding any interventions which could endanger her breast reconstruction [7]. Accordingly, the multidisciplinary team devised a personalized strategy, including radiation on the axilla and supraclavicular fossa, ECT on the breast skin and adjuvant systemic treatment with chemotherapy followed by endocrine manipulation.

**Figure 2:**
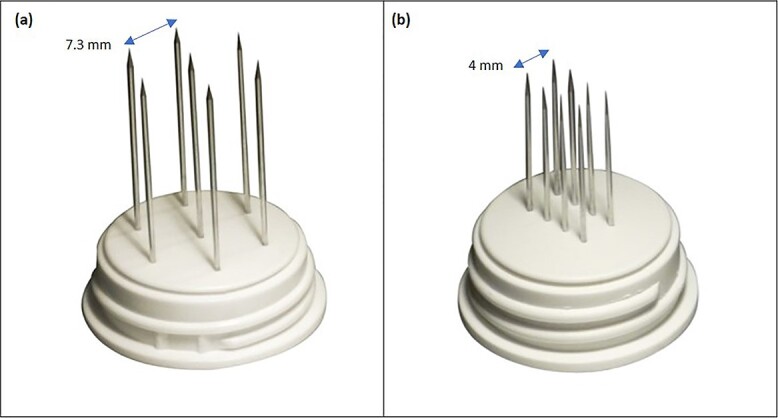
Needle electrode geometries: (**a**) hexagonal array; (**b**) linear array.

**Figure 3:**
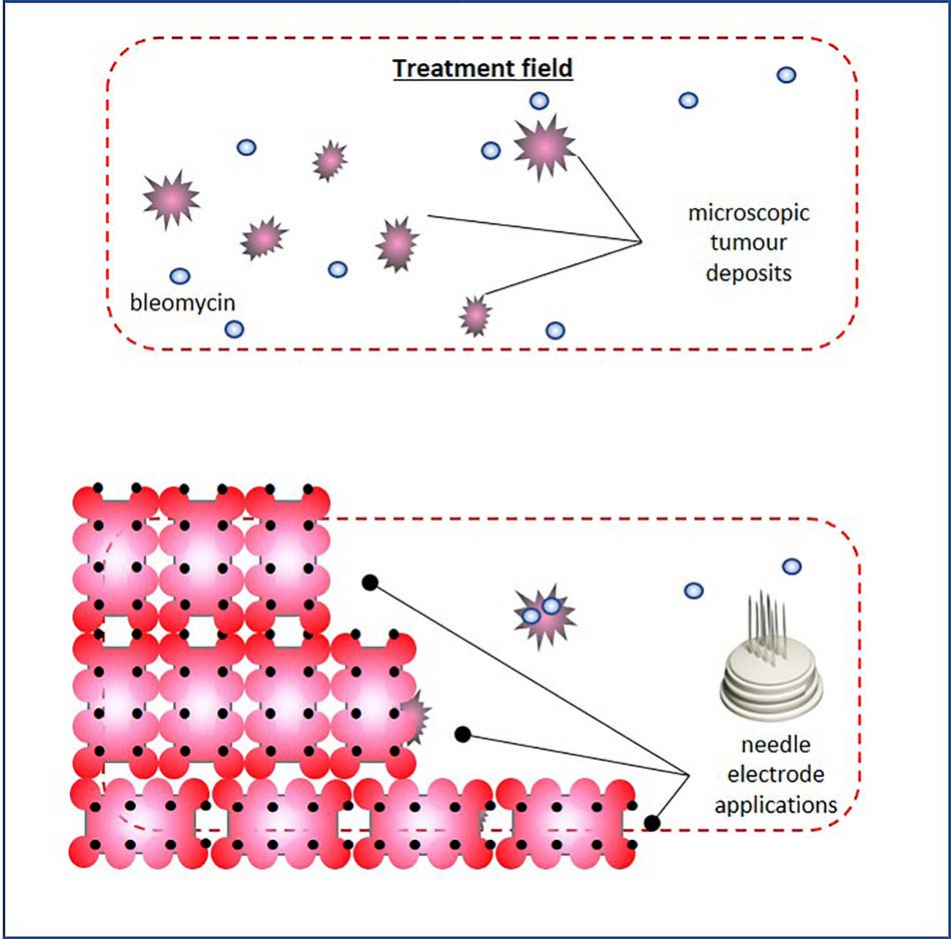
Treatment plan, and the electrochemotherapy field encompassed the skin around the previous surgical scar; following intravenous chemotherapy infusion, coverage of the treatment field was achieved through multiple juxtaposed insertions of a linear array needle electrode, and electrode overlapping or reapplication on the same area was carefully avoided to prevent tissue ischaemia; to avoid injuring the underlying prosthetic implant, the maximum needle depth did not exceed 5 mm.

**Figure 4:**
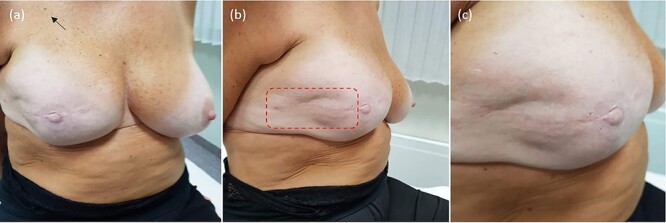
Treatment outcome; at 5-year follow-up, the patient is disease-free, with no evidence of local recurrence or long-term toxicity. The prosthetic implant remains *in situ*, providing satisfactory symmetry and aesthetic outcome: (**a**) overview; (**b**) treatment field of electrochemotherapy; (**c**) closeup on the treated skin.

The procedure lasted 20 minutes—under mild general sedation—and included a 17 000 IU i.v. bolus of bleomycin (dose deescalated from 15 000 to 10 000 IU/m^2^ to avert the risk of toxicity) [8]. A linear needle electrode was preferred as a pulse applicator to a hexagonal one because of its less invasivity and more controlled insertion (Fig. 2). The treatment field encompassed the previous surgical incision with a 3-cm width on each side; to this aim, we used multiple just-apposed electrode applications (Fig. 3). The postprocedural course was uneventful, and the patient was discharged on the following day with oral paracetamol as the sole medication. Locally, the skin was mildly erythematous but dry (Grade-1, according to the Common Terminology Criteria for Adverse Events [CTAE] v5.0) and was covered with silver sulfadiazine and a hydrogel dressing. There was no concerning increase in the inflammatory reaction in the following days, with complete resolution over the next few weeks. At 5 years, the patient remains disease-free, with preserved reconstruction (Fig. 4). She is monitored through regular physical examination and magnetic resonance.

## DISCUSSION

The advent of skin-sparing techniques with immediate reconstruction has brought an oncologically safe approach with superior cosmetic outcomes compared with ‘standard’ mastectomies. However, some concern persists related to the 3–4% risk of local relapse due to skin conservation [9]. The detection and management of locoregional recurrence require an appropriate physical examination, radiologic imaging and multidisciplinary evaluation because of its association with dismal prognosis. Clearly, ECT should not remedy suboptimal surgical treatment, and clinicians must aim for the best oncological radicality while preserving anatomical integrity. However, re-excision would ultimately cause excessive skin loss and implant removal in this patient. Therefore, ECT was agreed upon in the multimodal strategy with sparing intent.

**Figure 5:**
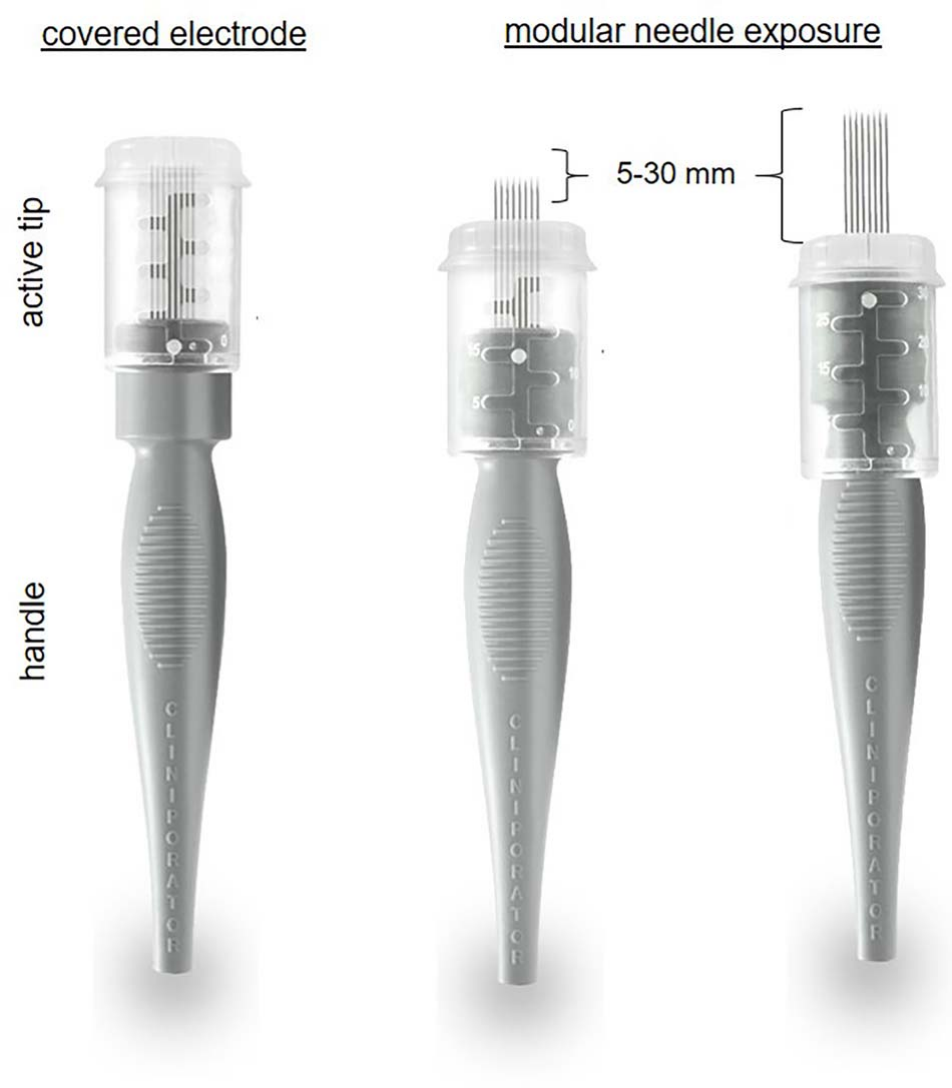
Example of variable-length needle electrode for precise tumour targeting.

Thanks to consistent efficacy across cancer types, ECT is increasingly appreciated by multidisciplinary cancer teams, mainly in Europe [10]. Albeit the impossibility to quantify its relative contribution in the absence of measurable disease in this case and given the systemic treatment received by the patient, it is worth noting that a highly effective therapy, associated with a nearly 50% chance of eradication [6], was applied safely. Avoiding skin toxicity was a primary concern because its potent antitumour activity could be a double-edged sword, leading to skin infection or ulceration (reported in up to 18% of patients) [11] and, ultimately, loss of the underlying implant. Furthermore, it has been reported that exposure of cells to electric pulses increases the cytotoxicity of bleomycin by ~8000-fold [12]. In this regard, the development of dedicated standard operating procedures, based on nearly three decades of basic science research, has permitted a safe translation of the procedure in clinical practice [10, 13]. The following factors may have favoured a positive outcome in our patient: (i) the excellent performance status, (ii) the absence of macroscopic disease and previous radiation, (iii) the reduced dose of bleomycin [8], (iv) the meticulous composition of the treatment field (Fig. 1) and (v) the small-size electrode. In this regard, thanks to recent technical advances, adjustable-length electrodes allow for modular needle exposure and more accurate treatment delivery (Fig. 5). Interestingly, ECT lends itself to intraoperative application, similar to radiotherapy. As such, it could be used following surgical resection to sterilize the tumour bed, an approach that could be advantageous in our case, allowing us to treat the skin flaps from within while avoiding the risk of injury to the prosthetic implant. Adopting the standard operating procedures along with careful patient selection, meticulous treatment delivery and attention to technical details to avert toxicity will be crucial for sharing clinical experiences and rigorous evaluation of this approach [14, 15].

In summary, this report illustrates the proof-of-concept of adjuvant ECT in breast cancer. As with any treatment, the benefits are higher when the indications are appropriate, and the quest for evidence basis should temper enthusiasm. Hence, multidisciplinary teams should refine selection criteria and pursue any strategy to minimize the risk of toxicity, all the more in the presence of a prosthetic implant.

## AUTHORS’ CONTRIBUTION

Study conception (L.G.C., N.B.), patient treatment (L.G.C., N.B., N.M.), data collection (N.B.), draft preparation (L.G.C.), manuscript revision and approval (L.G.C., N.B., N.M.).
